# Osmolarity and Glucose Differentially Regulate Aldose Reductase Activity in Cultured Mouse Podocytes

**DOI:** 10.1155/2011/278963

**Published:** 2011-12-29

**Authors:** Barbara Lewko, Elżbieta Latawiec, Anna Maryn, Anna Barczyńska, Michał Pikuła, Maciej Zieliński, Apolonia Rybczyńska

**Affiliations:** ^1^Department of Pathophysiology, Medical University of Gdansk, 80-211 Gdansk, Poland; ^2^Laboratory of Cellular and Molecular Nephrology, Mossakowski Medical Research Centre, Polish Academy of Sciences, 02-106 Warsaw/Gdansk, Poland; ^3^Department of Clinical Immunology and Transplantation, Medical University of Gdansk, 80-210 Gdansk, Poland

## Abstract

Podocyte injury is associated with progression of many renal diseases, including diabetic nephropathy. In this study we examined whether aldose reductase (AR), the enzyme implicated in diabetic complications in different tissues, is modulated by high glucose and osmolarity in podocyte cells. AR mRNA, protein expression, and activity were measured in mouse podocytes cultured in both normal and high glucose and osmolarity for 6 hours to 5 days. Hyperosmolarity acutely stimulated AR expression and activity, with subsequent increase of AR expression but decrease of activity. High glucose also elevated AR protein level; however, this was not accompanied by respective enzyme activation. Furthermore, high glucose appeared to counteract the osmolarity-dependent activation of AR. In conclusion, in podocytes AR is modulated by high glucose and increased osmolarity in a different manner. Posttranslational events may affect AR activity independent of enzyme protein amount. Activation of AR in podocytes may be implicated in diabetic podocytopathy.

## 1. Introduction

Aldose reductase (AKR1B1, EC.1.1.1.21, abbreviated as AR), a member of the aldoketo reductase superfamily, catalyzes the reduction of broad spectrum of aldehydes, using NADPH as a cofactor. Specific AR substrates comprise saturated and unsaturated aldehydes such as progesterone, isocorticosteroids, aldehydes derived from biogenc amines, methylglyoxal, and other harmful metabolites [1–3]. The enzyme is distributed with varying abundance within different tissues [4]. The diversity of AR substrate types and its wide tissue distribution indicates that one of the physiological roles of AR may be detoxification of endogenous and xenobiotic aldehydes. Further postulated roles of this enzyme include cell protection from oxidative and osmotic stresses [5]. The antioxidant defense involves reduction of highly reactive aldehydes produced by lipid peroxidation [6, 7] as well as the reduction of glutathione conjugates of unsaturated aldehydes [8]. The osmoprotective role of AR is associated with reduction of glucose to sorbitol whose intracellular concentration counteracts extracellular osmotic pressure. Physiological importance of this mechanism has been particularly well documented in renal medullary cells in which AR activity and protein synthesis were induced by high extracellular NaCl concentration [9, 10]. 

Glucose, with its apparent Km of 50–200 mM [3], is a poor substrate for AR. Abnormally high intracellular sugar is required to trigger aldose reductase pathway. In consequence, glucose is converted to sorbitol and further to fructose, with extensive NADPH consumption. Major cytotoxic effects of this pathway include oxidative stress induced by a diminished pool of GSH, intracellular sorbitol accumulation, and increased levels of fructose and its metabolites. Finally, evidence is growing that AR may be involved in inflammatory responses by affecting the NF-*κ*B-dependent expression of cytokines and chemokines [11, 12].

Within the kidney, the highest expression and activity of AR is found in the medullary region. In renal cortex abundance of the enzyme is very low as compared to medulla, as well as to various other tissues [4, 13]. Nevertheless, detailed studies have revealed the presence of AR in mesangial cells (MCs) and in podocytes of the glomeruli [14–16]. Increased expression of the enzyme has been demonstrated in the glomeruli of diabetic patients [17], while both mRNA and activity of AR were elevated in rat mesangial cells cultured in high glucose [18].

Whereas it is accepted that aldose reductase is implicated in pathogenesis of diabetic glomerulopathy [19–22], its role in podocytes has not yet been investigated. Podocytes are terminally differentiated cells, covering glomerular basement membrane with interdigitating foot processes connected by slit diaphragms. They play a critical role in maintaining the glomerular filter and in producing growth factors for both mesangial and endothelial cells. Due to their limited ability to proliferate and to replenish lost cells, podocyte impairment is considered to play a central role in the development of a majority of glomerular diseases [23]. In view of recent findings it seems likely that in diabetic patients the upregulation of AR pathway could contribute to deleterious changes in podocytes. The present study was designed to investigate the effect of high glucose and osmolarity, the two major factors affecting glomerular cells in diabetes, on the expression and activity of AR in podocytes.

## 2. Materials and Methods

### 2.1. Cell Cultures and Experimental Protocols

Conditionally immortalized mouse podocytes (Clone SVI, generous gift from Dr. N. Endlich, Greifswald University, Germany) were cultured as described previously [24]. Differentiated cells were grown in a standard RPMI1640 medium containing 5% FBS, 100 U/mL penicillin, and 0.1 mg/mL streptomycin (Sigma-Aldrich). At zero time podocytes were switched for 6, 12, 24, 48 hours, or 5 days to experimental media: NG-Nosm (normal glucose, normal osmolarity) containing 5.5 mM glucose and 285 mOsm/L, NG-Hosm (normal glucose, high osmolarity) containing 5.5 mM glucose and 385 mOsm/L, HG Nosm (high glucose, normal osmolarity) containing 30 mM glucose and 285 mOsm/L, and HG-Hosm (high glucose, high osmolarity) containing 30 mM glucose and 385 mOsm/L. Experimental media were based on RPMI1640 without glucose (Sigma-Aldrich) and osmolarity was adjusted by adding mannitol. All media were supplemented with 5% FBS and antibiotics, as indicated above. Osmolarity was checked using Vapor Pressure Osmometer (VAPRO 5520, Wescor Biomedical Systems, France). 

It has been shown previously that cultured human embryonic kidney (HEK) cells rapidly utilize glucose, which within 24 hours may result in glucose starvation, endoplasmatic reticulum stress, and underglycosylation of numerous proteins [25]. Therefore, using a glucometer (Accu-Chek, Roche Applied Science) we have estimated changes in glucose content in the NG and HG media from podocytes cultured for 0, 24, and 48 hours (Table 1). Based on the obtained results, 48-hour interval has been chosen for replacement of experimental media.

### 2.2. Isolation and Determination of Aldose Reductase Activity

At indicated time, culture flasks were placed on ice, experimental media were discarded, and 400 *μ*L ice-cold lysis buffer containing (in mM) 50 HEPES (pH 7.2), 2 DTT, 5 EDTA, and 1 tablet/10 mL protease inhibitor cocktail (Complete Mini, Roche Applied Science) was added to each flask. The cells were scrapped, transfered into eppendorf tubes, and centrifuged at +4°C, 14,000 rpm for 30 minutes (Eppendorf Centrifuge 5810R). Resulting supernatant was stored at −80°C, except for an aliquot of 25 *μ*L that was used for immediate protein assay according to the Bradford method [26]. Bovine serum albumin was used as a standard.

Activity of AR was determined spectrophotometrically (Ultrospec 3000) at 37°C as described previously [10]. In all tested groups, steady decrease in absorbance was observed up to 10 min after reaction was started (data not shown). Therefore, measurements were conducted for 6 minutes in the presence and absence of D,L-glyceraldehyde to correct for unspecific NADPH reductase activity [27]. AR activity was normalized to protein content and expressed in mU/*μ*g (nmol NADPH oxidized per minute per *μ*g protein), based on a molar absorption coefficient of 6220 M^−1^·cm^−1^.

### 2.3. Immunoblot Analysis

Immunobloting was performed using standard techniques. In brief, trypsynized cells were centrifuged at 2,000 rpm for 10 minutes in +4°C; resulting pellet was lysed on ice in a buffer (pH 8.0) containing 1% Nonidet P-40, 20 mM Tris, 140 mM NaCl, 2 mM EDTA, 10% glycerol, and protease inhibitor cocktail (Complete Mini, Roche Applied Science) and centrifuged for 20 minutes at 14,000 rpm in +4°C. Fifteen micrograms of total protein were subjected to SDS-PAGE and transfer electrophoresis. The proteins on PVDF Immobilion membranes (Millipore, Bedford, MA, USA) were probed with primary antibodies to AR (1 : 400, rabbit polyclonal, Santa Cruz Biotechnology Inc., Santa Cruz, CA, USA) and to *α*-smooth muscle actin (1 : 2000, mouse monoclonal, Sigma-Aldrich, St. Louis, MO, USA) followed by alkaline phosphatase-labeled secondary antibodies (goat anti-rabbit IgG and goat anti-mouse IgG, Santa Cruz Biotechnology Inc). The complexes were visualised with 5-bromo-chloro-3-indolyl phosphate (BCIP) and nitro blue tetrazolium (NBT; Sigma-Aldrich) and photographed in UVP BioImaging GDS-8000 system (UVP Inc., Upland, CA), using LabWorks 4.0 Image Acquisition and Analysis Software.

### 2.4. RNA Isolation and Reverse Transcription-Polymerase Chain Reaction (RT-PCR)

Total RNA was extracted from each culture sample using TRI Reagent (Sigma-Aldrich) according to the manufacturer's instructions. RNA purity and concentration were determined by measuring the optical densities at 260 nm and 280 nm. Two micrograms of RNA were reverse transcribed at 37°C for 60 min using 0.1 *μ*M antisense primer, 5 U/*μ*L MuLV reverse transcriptase (Promega, Madison, WI, USA ), 0.5 mM deoxynucleotide triphosphate mixture (dNTP), and 1 U/*μ*L RNase inhibitor (Sigma-Aldrich). The PCR step was performed using 2.5 U Maxima Hot Start Taq DNA Polymerase (Fermentas Int Inc.), with 20 *μ*L RT product and 80 *μ*L PCR mix containing 0.1 *μ*M sense primer. Primer sequences and PCR amplification conditions are shown in Table 2. Controls containing water in place of RNA were included in all experiments and gave negative results. The PCR products (18 *μ*L/lane) were resolved on ethidium bromide-stained 1.4% agarose gels, viewed and photographed on a UV transilluminator (UVP Inc., Upland, CA).

### 2.5. Densitometry

Semiquantitative analysis of the band optical densities was performed using Quantity One software (Bio-Rad, Hercules, CA, USA) and normalized to *β*-actin (RT-PCR) or to *α*-smoth muscle actin (immunobloting).

### 2.6. Flow Cytometry

Following incubations, the cells were washed with PBS, trypsinized, suspended in PBS, and centrifuged two times for 7 minutes at 400 × g at room temperature. The pellets were then resuspended in 1 mL of a cold permeabilizing buffer (Ebioscience, USA), incubated for 30 minutes in room temperature, centrifuged and washed with 1 mL washing buffer (WB) (Ebioscience, USA ). Finally, 1.5 × 10^5^ cells/tube were stained for 60 minutes at room temperature with 1 : 100-diluted rabbit antibodies to AR (Santa Cruz Biotechnology Inc., Santa Cruz, CA, USA). Antigen-bound antibodies were visualized by 40 minutes incubation with 1 : 100 diluted Alexa488-conjugated donkey anti-rabbit IgG (Molecular Probes, Eugene,OR, USA). Stained cells were washed with 1 mL WB, resuspended in 500 *μ*L PBS and analyzed in flow cytometer (Canto II Flow Cytometer, BD, USA). Basing on measurements of the single-cell fluorescence intensities, arbitrary selection of AR-positive cells was performed. For each selected population, the mean fluorescence index (MFI) was determined using FACS Diva software (BD, USA). Negative controls displayed negligible fluorescence of permeabilized podocytes.

### 2.7. Immunocytochemistry

For immunocytochemical analysis, the podocytes were grown on collagen-coated round glass cover slips in 24-well culture plates containing experimental media, as indicated before. Immunocytochemistry was performed as described previously [28]. Briefly, the cells were fixed (2% paraformaldehyde), permeabilized (0.3% Triton X-100), and incubated in blocking solution (2% fetal bovine serum, 2% bovine serum albumin, 0.2% fish gelatine, in PBS) for 60 minutes. This was followed by incubations, 60 min with primary rabbit antibody to AR (1 : 100, Santa Cruz Biotechnology, Inc.) and 45 min with secondary anti-rabbit IgG conjugated with Cy3 (1 : 200, Rockland Immunochemicals Inc Gilbertsville, PA, USA). Nonspecific staining was controlled by replacing primary antibody with blocking solution alone. F-actin was stained using 1 : 200 Alexa 488-phalloidin conjugate (Molecular Probes, Eugene, OR, USA). Apoptotic nuclei were detected by DNA condensation, using 1 *μ*g/mL 4′,6-diamidino-2-phenylindole dihydrochloride (DAPI, Merck Chemicals). All antibodies and stains were diluted in blocking solution. The cover slips were mounted on microscope slides using 15% Mowiol solution (Calbiochem, La Jolla, CA), and stained cells were analyzed under fluorescence microscope (Olympus IX51), using cellSens v.1.3 imaging software (Olympus).

### 2.8. Statistical Analysis

All of the data are presented as means ± SEM from 3 to 5 independent experiments. The statistical analyses were performed using SigmaStat (version 3.0. for Windows; SPSS Inc., Chicago, IL, USA). Data were analysed using Student's *t*-test for comparisons. Statistical significance is indicated by *P* < 0.05.

## 3. Results

### 3.1. Time Course of AR Activity in Podocytes

**Figure 1:**
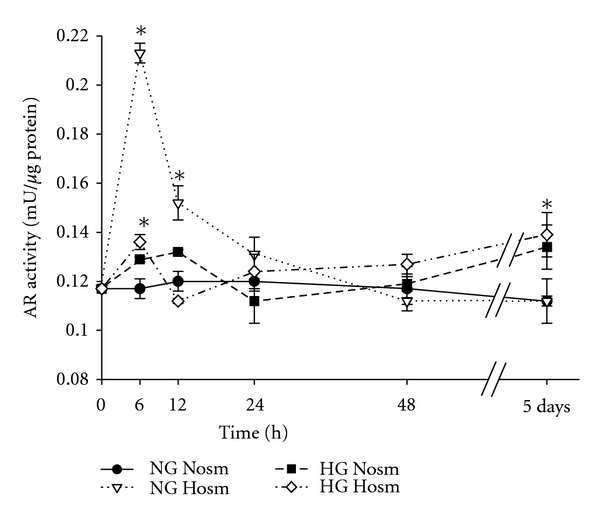
Time course of the changes in podocytic aldose reductase (AR) activity. Podocytes were cultured for indicated time periods in media with normal (5.6 mM, NG) or high (30 mM, HG) glucose content, and/or normal (285, Nosm) or high (385, Hosm) osmolarity. Cell lysates were prepared as indicated in Materials and Methods. Enzyme activities were calculated from the rate of the decrease in NADPH absorbance and are shown as absolute values. Data are expressed as means ± SE from 3–5 independent experiments, *P < 0.05 versus NG-Nosm cells.

In NG-Nosm cells, AR activity remained constant during the whole incubation time (Figure 1). In contrast, hyperosmolar conditions stimulated the activity of AR in NG cells reaching peak level (0.213 ± 0.004 versus 0.117 ± 0.004 mU/*μ*g in NG-Nosm, *P* < 0.05) already after 6 hours of incubation. Thereafter, a gradual decrease was observed, reaching the Nosm level after 48 hours. On the other hand, in HG medium after 6 hours only slight increase of AR activity was observed. After 5 days it reached the level that was only 1.2-fold higher than in the NG-Nosm group. Time course of changes in enzyme activities was similar in HG podocytes from both, normo- and hyperosmolar media which suggests that high glucose counteracted the stimulatory effect elicited by high osmolarity in NG cells.

### 3.2. Effect of Increased Osmolarity on AR Protein Expression and Activity

**Figure 2:**
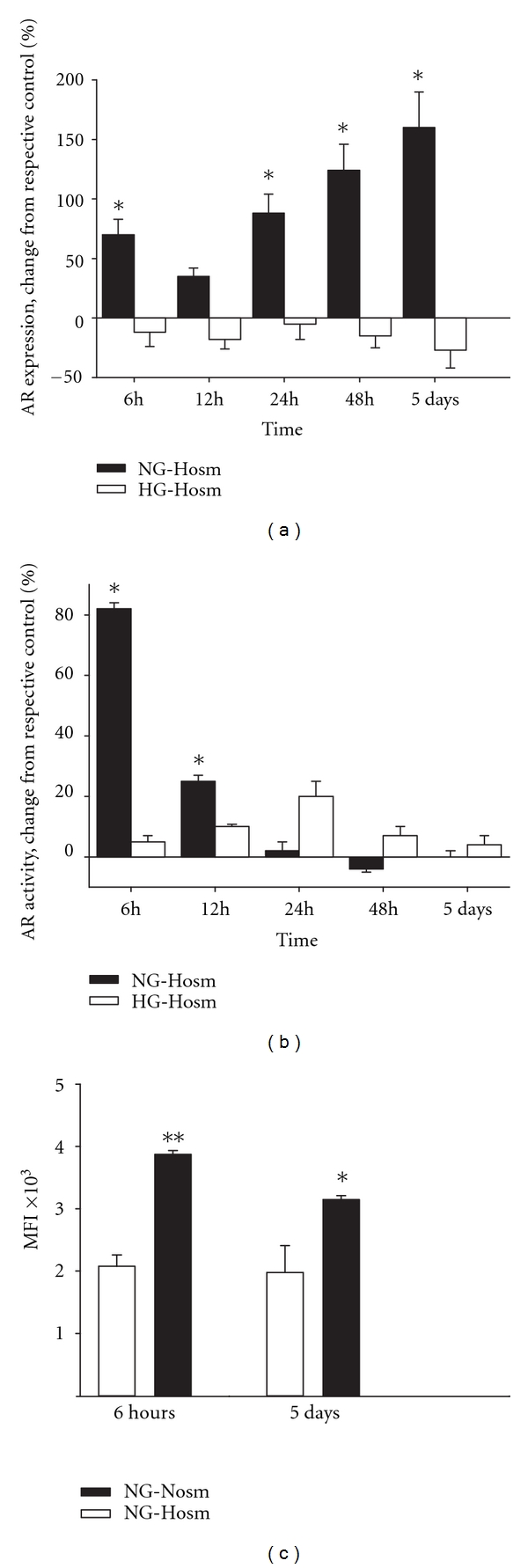
Effect of high osmolarity on (a, c) protein expression and (b) activity of AR in podocytes cultured in normal (NG) and high (HG) glucose. Protein expression was quantified by densitometry of immunoblot bands (a) and by flow cytometry (c) as indicated in Materials and Methods. Data are shown as percent changes of respective normoosmolar (Nosm) control values at the indicated time points (a, b) or as a mean fluorescence index, MFI (c). Results are expressed as means ± SE from 3–5 independent experiments, *P < 0.05, **P < 0.01 versus Nosm cells from respective groups.

Semiquantitative immunoblot analyses of the cells at each incubation time were performed to check whether observed changes in AR activity were related to changes in enzyme protein expression. The effects of short-time (6 hours) and prolonged (5 days) exposition to experimental conditions were further checked by flow cytometry. Densitometric quantification of obtained 37 kDa bands revealed that in hyperosmolar conditions, after 6-hour incubation AR expression in NG podocytes was markedly elevated (by 70 ± 13%, *P* < 0.05 versus NG-Nosm) (Figure 2(a)). Quantitative flow cytometric analysis (Figure 2(c)) yielded similar results, showing that AR increased by 86 ± 2% (*P* < 0.01). Respectively, activity of the enzyme increased by 82 ± 2%, *P* < 0.05 as compared to the NG-Nosm cells (Figure 2(b)). However, while AR protein expression in NG-Hosm cells remained increased during entire time activity of the enzyme dropped to the basal level after 24 hours. Conversely, no significant changes in expression or activity of AR were observed in HG podocytes exposed to increased osmolarity. 

**Figure 3:**
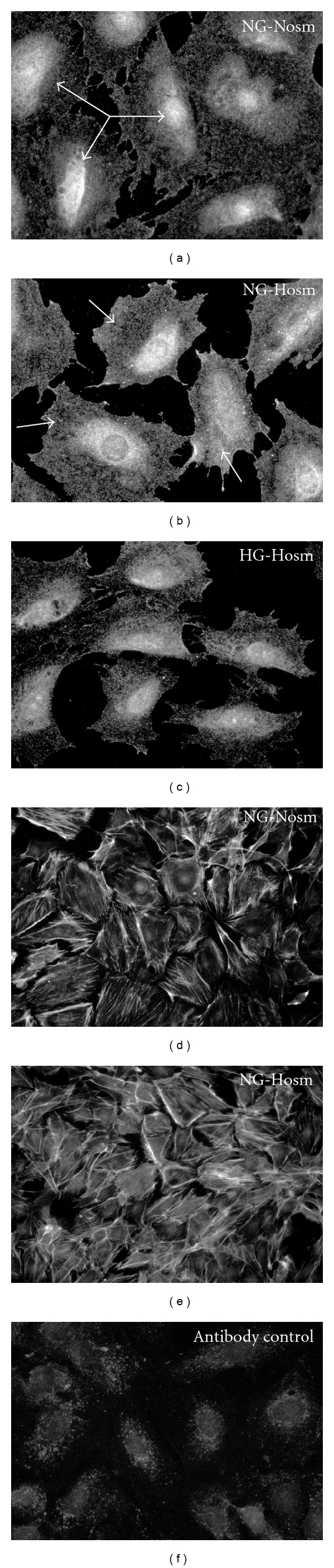
In podocytes cultured in normal glucose (NG), 5-day exposition to hyperosmolar conditions (Hosm) noticeably increased and redistributed AR immunostaining (b), as compared to the cells from normoosmolar (NG-Nosm) (a) or high glucose (HG-Hosm) (c) conditions. In the NG-Nosm cells, immunoreactivity was localized predominantly in perinuclear regions (arrows), while in NG-Hosm cells, intensive staining was diffuse throughout the cytoplasm (arrows). HG-Hosm cells stained to AR in a diffuse manner, with less pronounced perinuclear regions as compared to NG-Nosm. In response to high osmolarity, rearrangements of F-actin cytoskeleton were observed in NG cells. Thick transcellular stress fibers observed in NG-Nosm cells (d) were replaced by thin, more delicate F-actin filaments (e). Nonspecific staining control (f) was prepared as described in Materials and Methods. Magnification ×400 (a–c, f) and ×200 (d, e).

Immunofluorescent staining after 5 days of incubation confirmed that expression of AR in cytosol of NG-Hosm podocytes was upregulated, as compared to NG-Nosm and HG-Hosm cells (Figure 3(b)). NG-Nosm cells expressed evident perinuclear pools of AR with relatively weak staining in the rest of cytosolic regions (Figure 3(a)). In NG-Hosm podocytes, AR strongly stained in a diffuse pattern throughout the cell body. High osmolarity had no apparent effect on the F-actin cytoskeleton in NG cells. However, NG-Hosm podocytes displayed more delicate stress fibers, which was particularly noticeable after 5 days of incubation (Figure 3(d)). 

### 3.3. Effect of Glucose Concentration on AR Protein Expression and Activity

**Figure 4:**
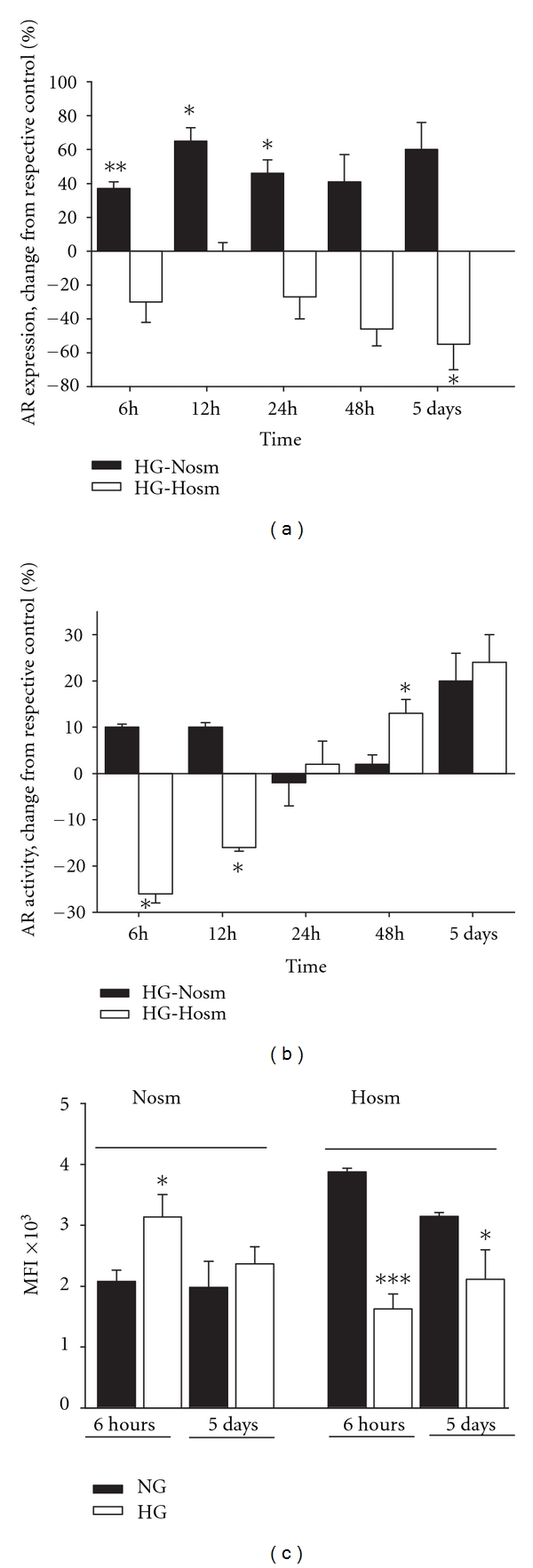
Effect of high glucose on (a, c) protein expression and (b) activity of AR in podocytes cultured in normo- (Nosm) and high- (Hosm) osmotic medium. Protein expression was quantified by densitometry of immunoblot bands (a) and by flow cytometry (c) as indicated in Materials and Methods. Data are shown as percent changes of respective normoglycemic (NG) control values at the indicated time points (a, b) or as a mean fluorescence index, MFI (c). Results are expressed as means ± SE from 3–5 independent experiments, *P < 0.05, **P < 0.01, ***P < 0.001 versus NG cells from respective groups.

In podocytes cultured in HG-Nosm media, AR protein was markedly increased (by 37 ± 4%, *P* < 0.025 versus NG-Nosm) as early as 6 hours after shifting to high glucose (Figures 4(a) and 4(c)). The enzyme remained elevated during the whole incubation period. However, this increase was not accompanied by significant changes in enzyme activity (Figure 4(b)). Conversely, in podocytes cultured in HG- Hosm media, high glucose suppressed the expression of AR and this effect could be observed over the whole period of incubation. After 5 days, AR protein decreased by 55 ± 15%, *P* < 0.05 as compared to NG-Hosm cells. During the first 12 hours of incubation in HG, activity of AR was also suppressed (by 26 ± 2%, *P* < 0.05 versus NG-Hosm at 6 h), while longer incubation seemed to reverse this effect.

### 3.4. Expression of AR mRNA in Podocytes

**Figure 5:**
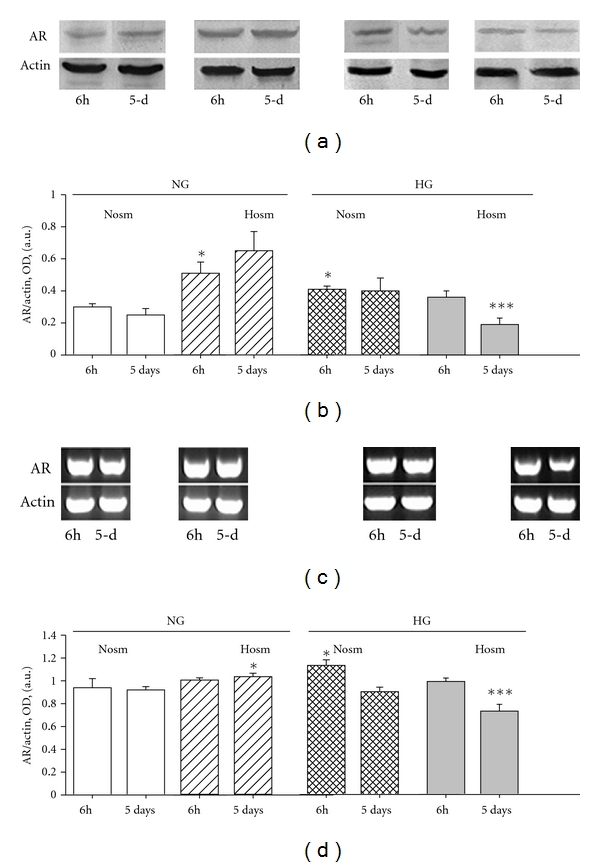
Expression of AR protein (a, b) and mRNA (c, d) in podocytes cultured in experimental media for 6 hours and 5 days, as described in Materials and Methods. (a) 15 *μ*g protein from cell lysates was immunoblotted using rabbit polyclonal anti-AR antibody and visualized with BCIP-NDT yielding ~37 kDa bands for AR and ~47 kDa bands for *α*-smooth muscle actin. (b) Results of densitometric quantification of corresponding immunoblot bands are expressed as optical density (OD) ratios of AR to actin bands. (c) 2 *μ*g total RNA was reverse transcribed and resulting cDNAs were amplified in 26 cycles, using specific primers for AR and for *β*-actin (see Table 2). 18 *μ*L of PCR products were electrophoresized in 1.4% agarose gel and stained with ethidium bromide. (d) Results of densitometric quantification of corresponding RT-PCR products are expressed as optical density (OD) ratios of AR to actin bands. *P < 0.05 versus NG-Nosm, **P < 0.05 versus NG-Nosm and HG-Nosm 5-d, ***P < 0.025 versus NG-Nosm and HG-Hosm 6 h. a.u., arbitrary units.

To verify whether the changes in enzyme protein expression reflect the mRNA levels for AR, we have reverse transcribed the total RNA from all experimental groups. Results of RT-PCR analysis revealed that already in basal (normal glucose and osmolarity) conditions, the expression of AR mRNA in podocytes was relatively high, with abundance comparable to that of glyceraldehyde-3-phosphate dehydrogenase (GAPDH, data not shown) and *β*-actin (Figure 5(c)). Since high ambient glucose concentration may affect the expression of GAPDH [29], for our further analyses we have used *β*-actin as a housekeeping gene. Comparison of optical density ratios (AR to actin) of respective immunoblots to the RT-PCR products showed that expressions of AR mRNA and protein were correspondingly modulated. Similar to the enzyme activity in NG-Nosm cells, AR mRNA and protein expression remained at a constant level up to 5 days of incubation. Enhanced (NG-Hosm and HG-Nosm cells) or suppressed (HG-Hosm cells) expression of AR protein (Figure 5(b)) was accompanied by respective changes in mRNA levels (Figure 5(d)). This may suggest that in our experimental conditions, transcription and translation processes were synchronized, while modulations of AR activity might be ascribed to posttranslational modifications of the enzyme.

## 4. Discussion

In the present study we show that in cultured mouse podocytes, expression and activity of aldose reductase (AR) are modulated by high glucose and hyperosmolarity in a different manner. High osmolarity alone induced a rapid but transient increase of AR activity (Figures 1, and 2(b)), while only slight activation of the enzyme by high glucose was observed. As compared to the NG-Hosm cells from respective incubation time points, during the first 12 hours of incubation activity of AR was even suppressed by high glucose (Figure 4(b)) with parallel decline in protein expression (Figures 4(a) and 4(c)). In a variety of cells, hyperglycemic conditions have been reported to upregulate aldose reductase activity both *in vivo* and *in vitro* [2, 30, 31]. Apart from numerous deleterious effects associated with its activation [5], the beneficial roles ascribed to AR include protection of the cells against glucose-derived osmotic [32] and oxidative [33, 34] stresses. Nevertheless, similar to results observed in our study, reduction of AR protein has been reported in mouse podocytes cultured in hyperosmolar HG medium for two weeks [35] and in arterial endothelium [36]. In turn, high glucose-induced loss of AR activity was found in epithelium from thick ascending limb of Henle's loop [37]. In our experiments, high glucose not only counteracted the activation of AR in HG-Hosm cells but also blunted the AR activity in HG-Nosm podocytes, despite upregulated enzyme protein (Figures 1, 4(a), and 4(c)). The mechanism underlying this paradoxical effect is not clear. It could be possible that AR activity was suppressed in response to intracellular sorbitol accumulation. However, while some authors have observed such suppression [9], others state that this enzyme does not exhibit feedback inhibition [38]. Another possibility is that glucose-induced cytotoxicity [39] that was observed in podocytes *in vivo* and *in vitro* [40] accounted for the drop in measured AR activity. In our experiments, however, nuclear DAPI staining did not reveal any signs of apoptosis in either of tested cell groups. It is also possible that in high glucose milieu the enzyme was affected by posttranslational modifications. One of the major molecular mechanisms implicated in hyperglycemia cell damage is induction of protein kinase C (PKC) isoforms [41]. Multiple interrelationships between PKC and AR have been described, including phosphorylation and translocation of AR to the mitochondria [42]. In high glucose milieu, PKC mediates also activation of NF-*κ*B, which, in turn, can regulate the transcription of AR gene [43]. Since PKC isoforms have been found in podocytes [44, 45], this mechanism could also be responsible for increased AR expression in HG-Nosm cells. On the other hand, enzymatic activity of AR is redox-sensitive due to the presence of cysteine residue (Cys-298) near the active site [46]. Both oxidation and S-thiolation of this residue modulate kinetics and activity of the enzyme [5, 47, 48]. It is therefore possible that high glucose-induced oxidative stress in podocytes [49, 50] could perturb the intracellular redox status leading to suppression of AR. Posttranslational modifications of AR may also involve an interplay between activatory S-nitrosylation of Cys-298 by nitric oxide (NO) and inhibitory glutathiolation of S-nitrosylated AR [51]. In addition, exposition to high glucose has been shown to inhibit NO synthesis *in vivo* and *in vitro* [52, 53]. Therefore, depletion of bioavailable NO could be another reason for loss of AR activity in podocytes incubated in HG medium. It has been reported previously that podocytes express neuronal [54] as well as inducible [55] forms of nitric oxide synthases; therefore such autocrine/paracrine regulation of AR by NO cannot be excluded. 

In contrast to high glucose, hyperosmolarity appeared to markedly stimulate AR activity in NG podocytes (Figures 1 and 2(b)). This was not surprising, as osmotic stress is considered to be a major regulator of AR expression, and aldose reductase is called “a hypertonicity stress protein” [56]. In the sequences of rat and human AR promoter regions, osmotic response element (ORE) sequences have been found [57, 58], and induction of aldose reductase in response to increased osmolarity was reported in numerous renal and nonrenal cells [10, 15, 59, 60]. Moreover, similar to podocytes, in renal tubular epithelium and in Schwann cells induction of AR occurred in response to osmotic stress but not to high glucose *per se* [61, 62]. However, in our experiments, the effect seemed to be relatively short-lived. Although AR protein increased with duration of incubation in the Hosm conditions (Figure 2), activity of the enzyme declined simultaneously. While positive correlation between AR expression and activity was observed in some cell types [62, 63], the discrepancies were found in other [64, 65] which suggests that activation of the enzyme by exogenous factors could play a role independent of protein level. There are two forms of native AR, one of them reduced (not active) and the other one oxidized (active) [66–69]. Since mannitol is known to exert antioxidant properties, prolonged incubation of podocytes in mannitol-containing hyperosmolar medium might favour the reduced form of the enzyme. It is also tempting to speculate that hyperosmolarity-induced intracellular glutathione depletion could stimulate a transmembrane cystine transport into the podocytes. Uptake of L-cystine in order to replenish intracellular glutathione levels has been shown in various cells and organs, including the kidney [70, 71]. However, cystine, by binding to Cys-298, could also directly inactivate aldose reductase [72]. Transmembrane cystine transport occurs either via glutamate transporters (Na^+^-dependent transport) or by glutamate/cystine antiporters [71] that are particularly abundant in brain tissue. By now, none of these transporters were found in podocytes. Nevertheless, podocytes have been shown to possess a differentiated amino acid transport system [73] including vesicular glutamate transporter [74, 75], and recent findings reveal more and more similarities between podocytes and neuronal cells. It seems therefore plausible that glutamate-dependent cystine transport may also be present in podocytes. However, this concept has not been proven yet. 

Relatively high basal AR mRNA expression (Figure 5(c)) may suggest that podocytes are well equipped to metabolize not only toxic aldehydes formed inside the cells but also diverse plasma-derived endogenous and xenobiotic aldehydes that may cross the cell membrane during filtration process. Prominent expression of AR protein has been previously observed in rat and ox podocytes [76, 77]. Such constitutive expression of the enzyme may allow the podocytes to quickly adjust their AR system accordingly to current solute composition and/or osmolarity. However, in hyperglycemia, modulation of AR expression and activity may contribute to the pathogenesis of glomerular impairment. In diabetes, increased plasma glucose results in elevated plasma osmolality. According to our HG-Hosm results, activity of podocytic AR may be then suppressed, which could impair antiosmotic defence and lead to podocyte loss. On the other hand, increased AR protein expressed by the podocytes appeared to be one of the major antigens in patients with membranous nephropathy [16]. Therefore, osmolality-dependent elevation of AR protein in podocytes may trigger the inflammatory response within the glomerulus. Aldose reductase has also been shown to modulate production of cytokines such as tumor necrosis factor *α* (TNF *α*) or vascular endothelial growth factor (VEGF) in the kidney and vascular smooth muscle cells [5, 21, 48, 78]. In glomeruli, podocytes are a prominent source of VEGF [79], TNF *α* [80], as well as of other cytokines. Their expression not only contributes to the podocyte integrity and survival but also regulates the functions of other glomerular and tubular cells. It seems likely that in diabetic conditions, modulation of AR in podocytes may be essential for production of factors responsible for regulation of glomerular barrier structure and permeability. Additionally, nonimmune effects of AR should be considered, such as oxidative damage, sorbitol accumulation, or overproduction of fructose [5, 48].

In summary, these results show that in cultured podocytes aldose reductase is regulated by both high glucose and high osmolarity. Furthermore, changes in AR protein expressed by the cells do not necessarily correspond to changes in activity of the enzyme. High glucose alone elevated AR protein level, which was not accompanied by respective enzyme activation. High osmolarity in turn stimulated both activity and expression of AR in podocytes already within the first hours of exposition. However, while AR protein remained increased over the whole time of incubation, the activity of the enzyme concomitantly decreased. This suggests that in podocytes posttranslational events may affect AR activity independent of the enzyme protein content. Modulation of AR-dependent metabolic pathway in podocytes may be implicated in the pathomechanisms of diabetic podocytopathy.

## Figures and Tables

**Table 1:** Estimation of glucose content (mM) in the media from podocytes cultured for 0, 24, and 48 hours in the presence of normal (NG) and high (HG) glucose. Results are expressed as means ± SE from 5 independent measurements.

	0 hrs	24 hrs	48 hrs
NG	5.8 ± 0.2	5.0 ± 0.4	4.7 ± 0.5
HG	30.0 ± 0.4	27.3 ± 0.9	26.7 ± 1.3

**Table 2:** PCR conditions for aldose reductase and *β*-actin genes. Previously published primer sequences were verified by NCBI BLAST. Amplification was conducted for 26 cycles.

Gene	Sequence of primers (5′ to 3′)	Amplicon (bp)	Annealing temp. (°C)
Aldose reductase	Sense: CCCAGGTGTACCAGAATGAGA	580	53.1
	Antisense: TGGCTGCAATTGCTTTGATCC		

*β*-actin	Sense: CCGTAAAGACCTCTATGCCA	299	50.8
	Antisense: AAGAAAGGGTGTAAAACGCA		
